# The readiness of the Asian research ethics committees in responding to the COVID-19 pandemic: A multi-country survey

**DOI:** 10.12688/f1000research.143138.1

**Published:** 2024-01-08

**Authors:** Juntra Karbwang, Cristina E. Torres, Arthur M. Navarro, Phanthipha Wongwai, Edlyn B. Jimenez, Yashashri Shetty, Sudha Ramalingam, Paresh Koli, Lisa Amir, Septi Dewi Rachmawati, Monalisa Waworundeng, Harnawan Rizki, Asyraf Syahmi Mohd Noor, Prakash Ghimire, Pradip Gyanwali, Subhanshi Sharma, Namita Ghimire, Chandanie Wanigatunge, Kwanchanok Yimtae

**Affiliations:** 1 Drug Discovery and Development Center, Thammasat University, Pathum Thani, Bangkok, 12120, Thailand; 2 Clinical Coordination and Training Center, Strategic Initiative in Developing Capacity in Ethical Review (SIDCER), Thammasat University, Pathum Thani, 12120, Thailand; 3 National Institutes of Health, University of the Philippines Manila, Manila, Metro Manila, 1000, Philippines; 4 Clinical Coordination and Training Center, Forum for Ethical Review Committees in Asia and the Pacific (FERCAP), Thammasat University, Pathum Thani, 12120, Thailand; 5 Department of Social Sciences, University of the Philippines Manila, Manila, Metro Manila, 1000, Philippines; 6 Faculty of Medicine, Khon Kaen University, Nai Mueang, Khon Kaen, 40002, Thailand; 7 Department of Pharmacology & Therapeutics, Seth Gordhandas Sunderdas Medical College and King Edward Memorial Hospital, Mumbai, 400012, India; 8 Department of Community Medicine, PSG Institute of Medical Sciences and Reseach, Coimbatore, 641004, India; 9 Forum of Indonesian Recognized Ethics Committees (FIRREC), Jakarta, 10430, Indonesia; 10 Dental Research Ethics Committee Faculty of Dentistry, Universitas Indonesia, Jakarta, Jakarta, 10430, Indonesia; 11 Health Research Ethics Committee, Faculty of Medicine, Universitas Brawijaya, Malang, East Java, 65145, Indonesia; 12 Ethics Committee, Mochtar Riady Institute for Nanotechnology (MRIN), Tangerang, 15810, Indonesia; 13 National Institutes of Health, Ministry of Health, Federal Government of Malaysia, Kuala Lumpur, 40170, Malaysia; 14 National Ethical Review Board, Nepal Health Research Council, Ministry of Health & Population, Kathmandu, 7626, Nepal; 15 Faculty of Medical Science, University of Sri Jayewardenepura, Gangodawila, Nugegoda, 10250, Sri Lanka

**Keywords:** COVID-19, Pandemic, IRB, Institutional review board, research ethics committee, Strategic Initiative for Developing Capacity in Ethical Review, SIDCER, Network

## Abstract

**Background:**

COVID-19 is a highly challenging infectious disease. Research ethics committees (RECs) have challenges reviewing research on this new pandemic disease under a tight timeline and public pressure. This study aimed to assess RECs’ responses and review during the outbreak in seven Asian countries where the Strategic Initiative for Developing Capacity in Ethical Review (SIDCER) networks are active.

**Methods:**

The online survey was conducted in seven Asian countries from April to August 2021. Two sets of online questionnaires were developed, one set for the chairs/secretaries and another set for the REC members.

The REC profiles obtained from the REC members are descriptive in nature. Data from the chairs/secretaries were compared between the RECs with external quality assessment (SIDCER-Recognized RECs, SR-RECs) and non-external quality assessment (Non-SIDCER-Recognized RECs, NSR-RECs) and analyzed using a Chi-squared test.

**Results:**

A total of 688 REC members and 197 REC chairs/secretaries participated in the survey. Most RECs have standard operating procedures (SOPs), and have experience in reviewing all types of protocols, but 18.1% had no experience reviewing COVID-19 protocols. Most REC members need specific training on reviewing COVID-19 protocols (93%). In response to the outbreak, RECs used online reviews, increased meeting frequency and single/central REC. All SR-RECs had a member composition as required by the World Health Organisation ethics guidelines, while some NSR-RECs lacked non-affiliated and/or layperson members. SR-RECs reviewed more COVID-related product development protocols and indicated challenges in reviewing risk/benefit and vulnerability (0.010), informed consent form (0.002), and privacy and confidentiality (P = 0.020) than NSR-RECs.

**Conclusions:**

Surveyed RECs had a general knowledge of REC operation and played a significant role in reviewing COVID-19-related product development protocols. Having active networks of RECs across regions to share updated information and resources could be one of the strategies to promote readiness for future public health emergencies.

## Introduction

COVID-19 is a new, highly infectious disease that seriously challenges the research community. ^1^^,^^2^ There are limited effective drugs and vaccines available and, to date, very limited specific treatment for mild COVID-19, which accounts for 80% of COVID-19 infected patients. ^3^^,^^4^ Research on new treatments, prophylactics, and diagnostic products to address disease outbreaks is needed. ^3^^,^^5^ Under the limited supply of effective drugs and vaccines, flattening the curve of the infection depends on adherence to the public health socio-behavioural interventions imposed by the state. ^6^^,^^7^ Research on the effectiveness and acceptability of these non-pharmaceutical interventions is important to assist in making the decision about how to address the outbreak. ^8^^,^^9^ While the researchers are finding solutions to end the epidemic, the institutional review boards (IRBs)/research ethics committees (RECs) are responsible for the oversight of research conduct to ensure that the researchers adhere to scientific principles and uphold the ethical principles of autonomy, beneficence, and justice and that they are responsive to the COVID-19 outbreak. ^10^ Local IRBs/RECs have to encounter new challenges, some related to research approval of new investigational tools and approaches against the pandemic under a tight timeline and public pressure, ^1^^,^^2^ and others related to delays in ongoing activities due to the diversion of human resources towards the pandemic response. ^11^ Some local RECs in different countries may not be ready to address the challenges arising from the COVID-19 pandemic. ^12^

The present study aimed to find the RECs’ responses and review practices during the COVID-19 outbreak in Asian countries, where Strategic Initiative for Developing Capacity in Ethical Review (SIDCER) networks are active in training and quality evaluation of RECs ( www.SIDCER-FERCAP.org). This study describes how the local RECs in Asian countries faced and addressed the challenges in ethics review during the COVID-19 outbreak. Specifically, the study assessed RECs’ operational readiness and challenges to conduct the ethics review during the COVID-19 pandemic.

## Methods

A multi-center cross-sectional, descriptive survey was conducted in seven countries by local team members of the Forum for Ethical Review Committees in the Asian and Western Pacific Region (FERCAP) network. The survey was carried out from April to August 2021. FERCAP conducted a common online survey of RECs in Asia among the RECs with external quality assessment (SIDCER recognized REC (SR-REC)) and no external quality assessment (Non-SIDCER recognized REC (NSR-REC)) to determine the response and review practices of RECs during the COVID-19 outbreak.

### Study materials

Two sets of questionnaires were developed using online Google forms, one set for the chairs/secretaries, and another set for the members. The questionnaires for the chairs/secretaries focused on REC profiles, activities during the COVID-19 outbreak, and identification of training needs among SR-RECs and NSR-RECs. The questionnaires to be completed by members gathered information on the REC’s membership profile, number of protocols reviewed, challenges encountered during COVID-19, training needs, and their knowledge and attitudes about reviewing COVID-19 related research. Knowledge and attitude questions were also prepared on five Likert scales. The initial draft questionnaire was pretested by sending it to focal persons in each country to determine if there was a common understanding of the questions and the expected response. Several meetings were held to discuss and share specific country issues related to the survey instrument and the target population, and the protocol was revised accordingly. No reliability test was done.

### Study populations and sample size determination

The study enrolled REC chairs/secretaries and members in seven countries: India, Indonesia, Malaysia, Nepal, Philippines, Sri Lanka, and Thailand. The current project utilized the resources and infrastructure of the existing networks (FERCAP and local ethics networks of each country). FERCAP gathered information from RECs that are network members and from other RECs who would agree to respond to the survey. FERCAP targeted a purposive sample of 500 REC members. The recruitment was conducted from April to August 2021.

### Study procedure and data collection

A round of meetings was held among the involved team members to finalize the protocol and share the protocol along with the set of questionnaires and informed consent forms in the online Google form. After getting the approval from the REC, the selected chair/secretaries and the members were sent the Google form for their response.

### Ethical consideration

The study protocol was reviewed and approved by local RECs and some RECs granted exemption or subjected it to expedited review before the conduct of the survey in each country: Khon Kaen University Ethics Committee, Thailand, dated 31 March 2021 (HE641148); Republic of the Philippines, Department of health, Manila, Philippines, Single joint research ethics board, 1 June 2021 (SJREB-2021-40); Nepal Health Research Council, 24 March 2021 (190/2021P); Ethic Review committee Sri Lanka Medical Association, 21 May 2021 (ERC 21-010); Seth GS Medical College and KEM Hospital, Mumbai, India, 15 June 2021 (IEC (II)/OUT/427/2021); National Institute of Health (NIH), Malaysia, Medical research & Ethic Committee, 2 June 2021 (NMRR-21-969-59930 (IIR)); Faculty of Dentistry, University of Indonesia, Dental Research Ethics Committee, 5 April 2021 (01 Ethical exempted/FKGUI/IV/2021). ^13^ Written informed consent was obtained through voluntary action by answering the Google form questionnaire in English. The answers from the REC chairs/secretaries were identifiable. However, in the case of REC members, answers were anonymous.

### Data analysis

The collected data were analyzed to identify the common trends, significant challenges, and solutions. The information obtained from the REC members was described and presented in bar/pie/stacked charts. The data for the knowledge and attitude of REC members were presented in the stacked chart as a percentage of each value from the Likert scale; the data were also analyzed to obtain the means (SD) and interquartile range. The data from the chairs/secretaries were compared between SR-RECs and NSR-RECs presented in percentages and analyzed using the Chi-squared test in SPSS version 22.

## Results

The questionnaires were sent to 332 RECs, and the total responses from the chair/secretaries were 197 (59.3%), with 688 responses from the REC members ( Table 1).

**Table 1.** Number of research ethics committee (REC) questionnaires sent and responses from REC chairs/secretaries and members.

Country	REC questionnaires sent (n)	REC chair/secretary responded (n)	% of REC responded	REC members responded (n)
India	40	25 (11/14)	62.5%	84
Indonesia	14	11	78.6%	100
Malaysia	15	10	66.7%	84
Nepal	52	48	92.3%	108
Philippines	79	54	68.4%	125
Sri Lanka	10	10	100%	79
Thailand	122	39	32%	108
**Total**	**332**	**197**	**59.3%**	**688**

### Information from REC members survey

Fifty-one percent of the participants came from academic research institutions, followed by 33% from hospitals (Underlying data: Supplementary Figure 1 ^13^). The majority of participants were medical doctors/dentists, followed by other medical professionals such as nurses, pharmacists, etc. (Underlying data: Supplementary Figure 2 ^13^). There were equal numbers of social and biomedical scientists (7%), and a few laypersons and lawyers (6% and 3%, respectively).

Most members reviewed only scientific/technical issues and less than 20% of members reviewed only Informed Consent Forms (ICFs); however, 8.9% claimed that they reviewed both technical and ICF issues (Underlying data: Supplementary Figure 3 ^13^).

### Standard operating procedures (SOPs)

All of the countries have standard operating procedures (SOPs) but not all RECs in Sri Lanka had SOPs (Eight participants reported no SOPs) (Underlying data: Supplementary Figure 4 ^13^). Within those RECs that reported having SOPs, there was a lack of some standard procedures. A total of 12.5% of participants indicated no SOP on structure and composition, 11.8% on initial review, 19.1% on post-approval process and documentation, 19.7% on standard assessment, 18.1% on agenda and minutes, 30.6% on serious adverse events (SAE) review, and 30.6% on archiving. A total of 31.8% of participants indicated that the list of international and national guidelines was missing.

### Frequency of meeting (N = 681)

Most RECs had monthly board meetings (65.2%), followed by every two weeks (12.2%), and every week (7.6%) (except Sri Lanka), 9.1% quarterly, 2.5% every semester (except Sri Lanka), 1.0% annually (only in Malaysia and India). Only 2.3% had no REC meetings (only in Malaysia) (Underlying data: Supplementary Figure 5 ^13^).

### Participation of members in a different type of protocol review as part of their regular review function

Generally, members from all countries (N = 678) have experience in reviewing all types of protocol (Underlying data: Supplementary Figure 6 ^13^) but were most familiar with clinical research (79.9%) followed by public health (72.6%), socio-behavioural research (61.1%) and laboratory research (50.0%).

One hundred and twenty-four participants (18.1%) indicated that they had not been reviewing COVID-19 protocols. Generally, when reviewing COVID-19 protocols, members reviewed all elements required by international standards except advertisements, where half (50.6%) of the participants indicated that they had not reviewed this element (Underlying data: Supplementary Figure 7 ^13^).

### Challenges when reviewing COVID-19 protocols

For those members who reviewed COVID-19 protocols (N = 503), several challenges were identified with the top three being the review of risk/benefit (46.1%), scientific design (35.6%), and vulnerability 34.6%. However, one-third (29.2%) of participants reported that they had no difficulty in reviewing COVID-19 protocols (Underlying data: Supplementary Figure 8 ^13^).

### Training needs

A total of 87.0% of participants (N = 684) indicated the need for training when reviewing COVID-19 protocols. The top three training needs (identified from 678 participants) were ethical issues relating to clinical research (60.9%), risk/benefit assessment (57.1%), and international guidelines and regulations (55.0%) (Underlying data: Supplementary Figure 9 ^13^).

### Perspectives of REC members on the operation of RECs during the COVID-19 outbreak

**Figure 1.**
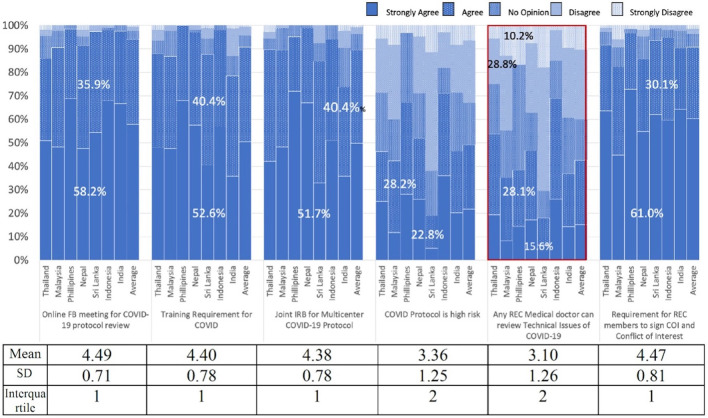
The perspectives of members on the operation of research ethics committees using a Likert Scale.

Regarding the review of COVID-19 protocols, most of the participants conducted an online full board meeting (94.1%) and used a joint or central REC review in the case of a multi-centred study (92.1%). The participants also agreed that RECs need training in ethics review of COVID-19 protocols (93%). The participants considered that the majority of COVID-19 protocols were high risk and should be reviewed by the full board. The majority of participants recognized the issues of confidentiality and conflicts of interest (CoI) involving COVID-19 protocols (91.1%) and the importance of addressing these issues ( Figure 1).

### Knowledge of REC members

Figure 1 demonstrates that more than 90% of the participants agreed that COVID-19 patients were vulnerable. They also agreed on the need for RECs to review and approve studies related to traditional medicine (82.2%), or unproven drugs, vaccines, and interventions for COVID-19 (85.1%). More than half of the participants thought that COVID-19 patients in hospitals should be allowed to participate in clinical trials. More than half of the participants were against employees of drug companies acting as principal investigators in Phase III clinical trials.

**Figure 2.**
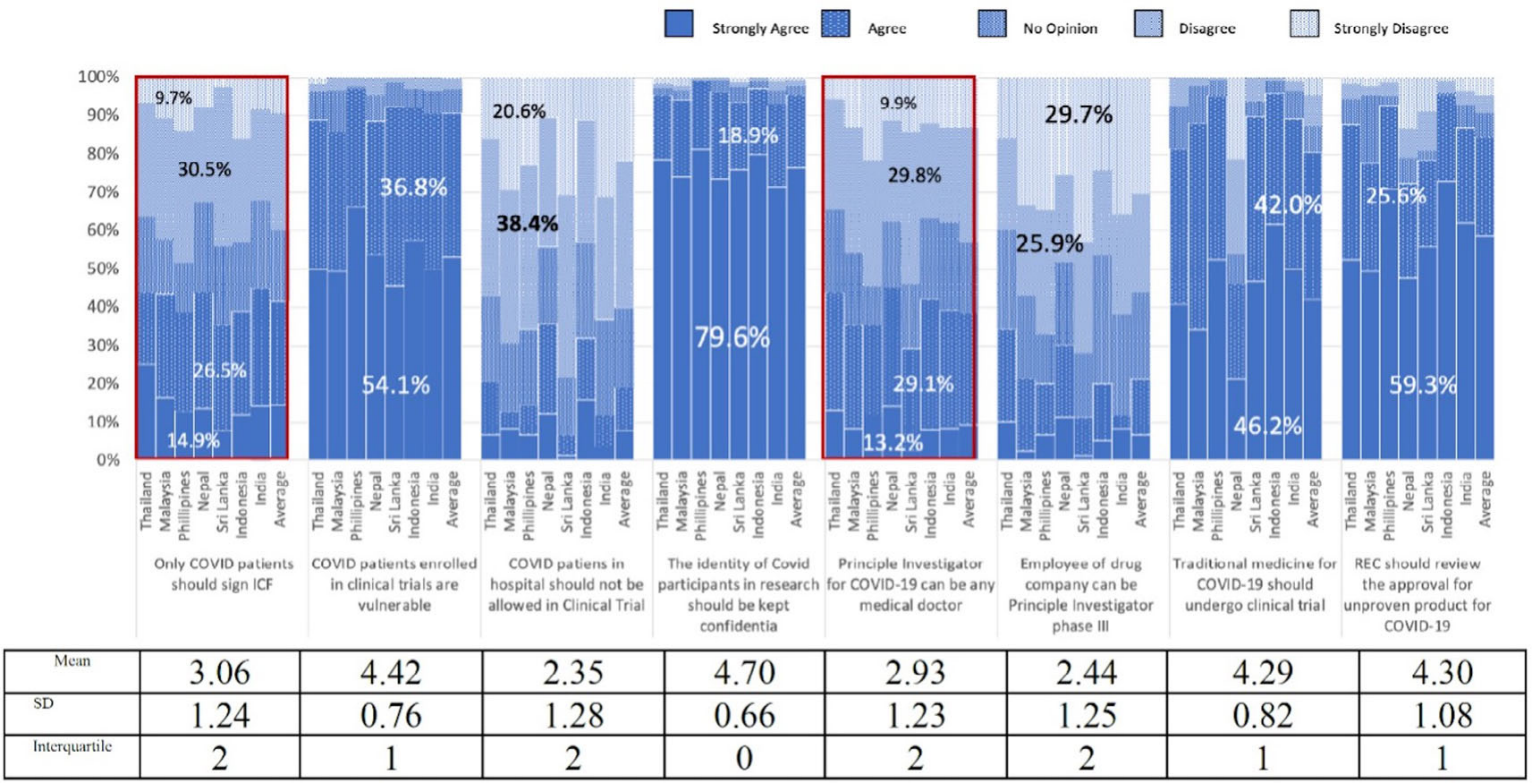
Perspectives of research ethics committee members towards COVID-19 protocol review.

There were controversial opinions on three items with regard to COVID-19 studies ^1^: The principal investigator can be any medical doctor, ^2^ Any REC medical doctor can review technical issues, and ^3^ Only COVID-19 patients should sign the ICFs ( Figures 1 and 2).

### SIDCER-recognized (SR) and non-SIDCER-recognized (NSR) RECs: Information obtained from chairs/secretaries

During the COVID-19 outbreak, similar measures were implemented in both SR-RECs and NSR-RECs surveyed such as masks, social distancing, community lockdown, travel restrictions, and limited access to institution facilities. The only difference between the SR-RECs and NSR-RECs was the limited access to institution facilities, which was found to be higher in SR-RECs (P = 0.03).

### Composition of RECs

**Figure 3.**
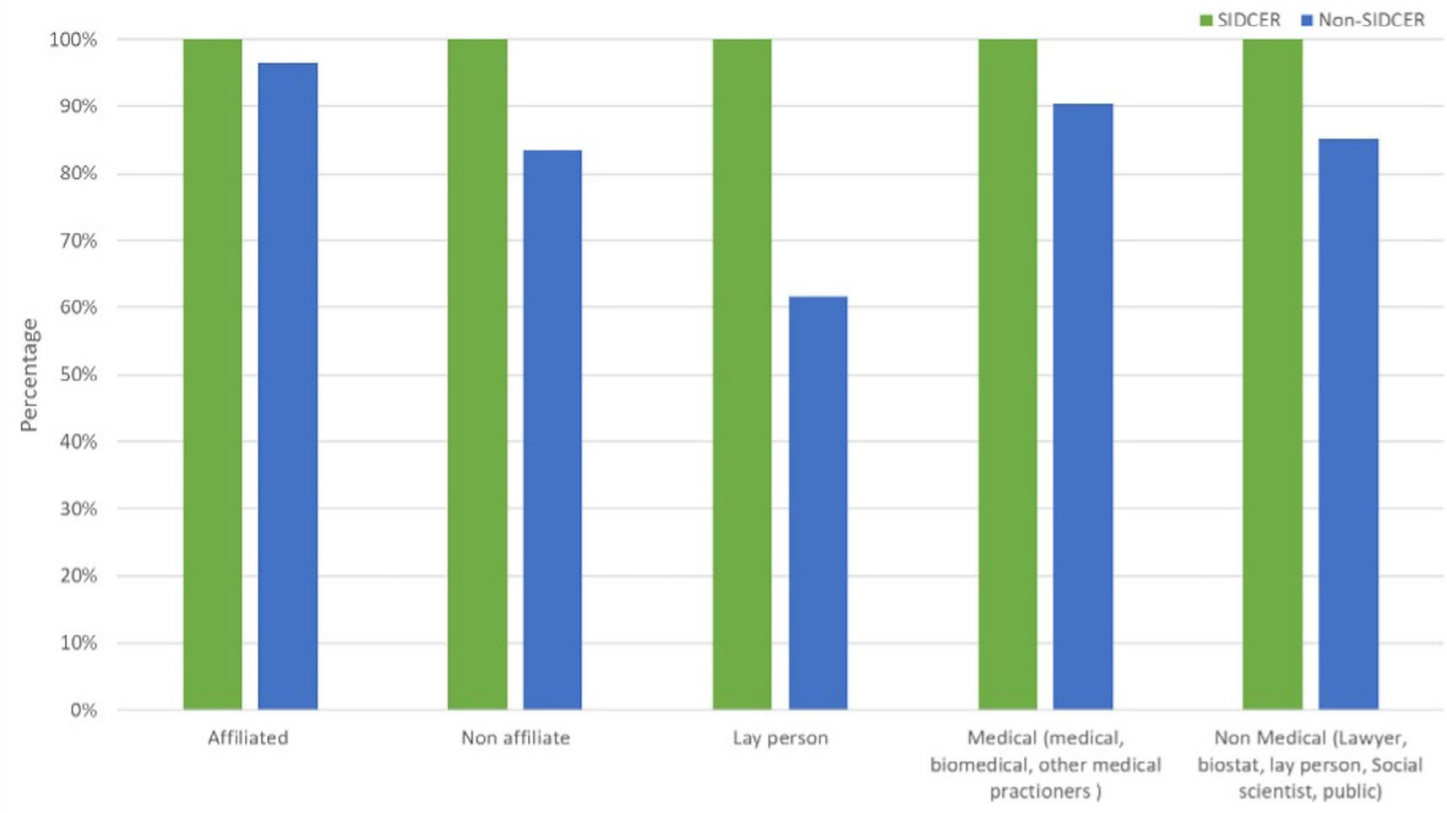
Composition of research ethics committees.

SR-RECs complied with the member composition of RECs as required by the regulatory requirements and international guidelines, while some of the NSR-RECs lack non-affiliated and lay members. This aspect was significantly different between SR-RECs and NSR-RECs (P = 0.000 and 0.000, respectively) ( Figure 3).

### Protocol review: REC meeting, the workload of the RECs, type of protocol review, and post-approval review

Both SR-RECs and NSR-RECs had adopted online meetings (80% and 66%, respectively). Some used online and face-to-face meetings (15% and 24%, respectively), and a few used face-to-face meetings only (5% and 10%, respectively).

**Figure 4.**
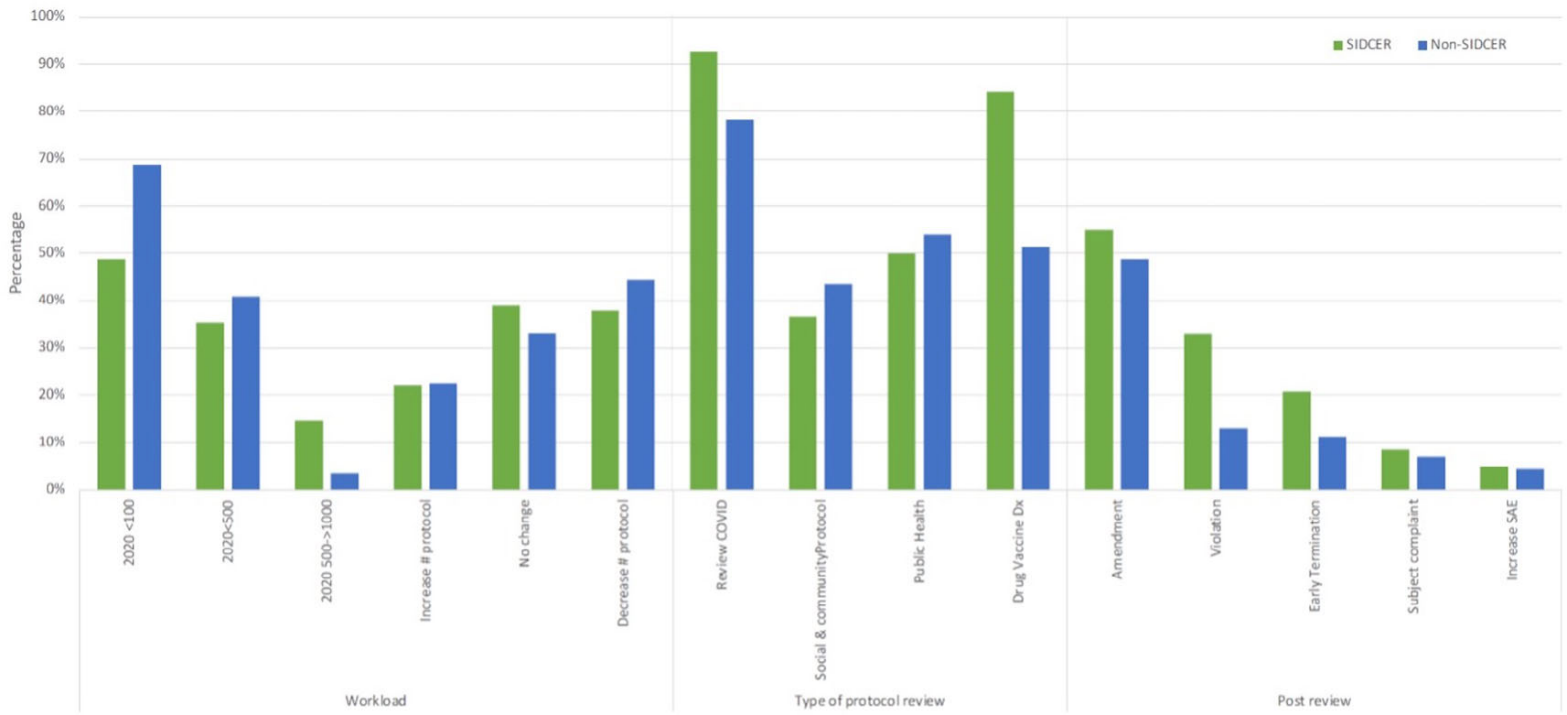
Number and type of protocol reviewed.

Figure 4 shows the workload of RECs, type of protocol reviewed, and post-approval review. More NSR-RECs reviewed less than 100 protocols during 2020 (P = 0.004) while more SR-RECs reviewed more than 500 protocols during 2020 (P < 0.004).

Both SR-RECs and NSR-RECs reviewed social and community, public health, and product development protocols. All countries reviewed COVID-19 protocols; however, some RECs did not review COVID-19 protocols (6 SR-RECs and 25 NSR-RECs). Significantly more SR-RECs reviewed COVID-19 protocols (P = 0.003) and product development protocols i.e. drug, vaccine, and diagnostic protocols (P = 0.000).

Both SR-RECs and NSR-RECs reviewed post-approval protocols. However, more SR-RECs reported having reviewed protocol violations (P = 0.001).

### Challenges in reviewing COVID-19 protocols

**Figure 5.**
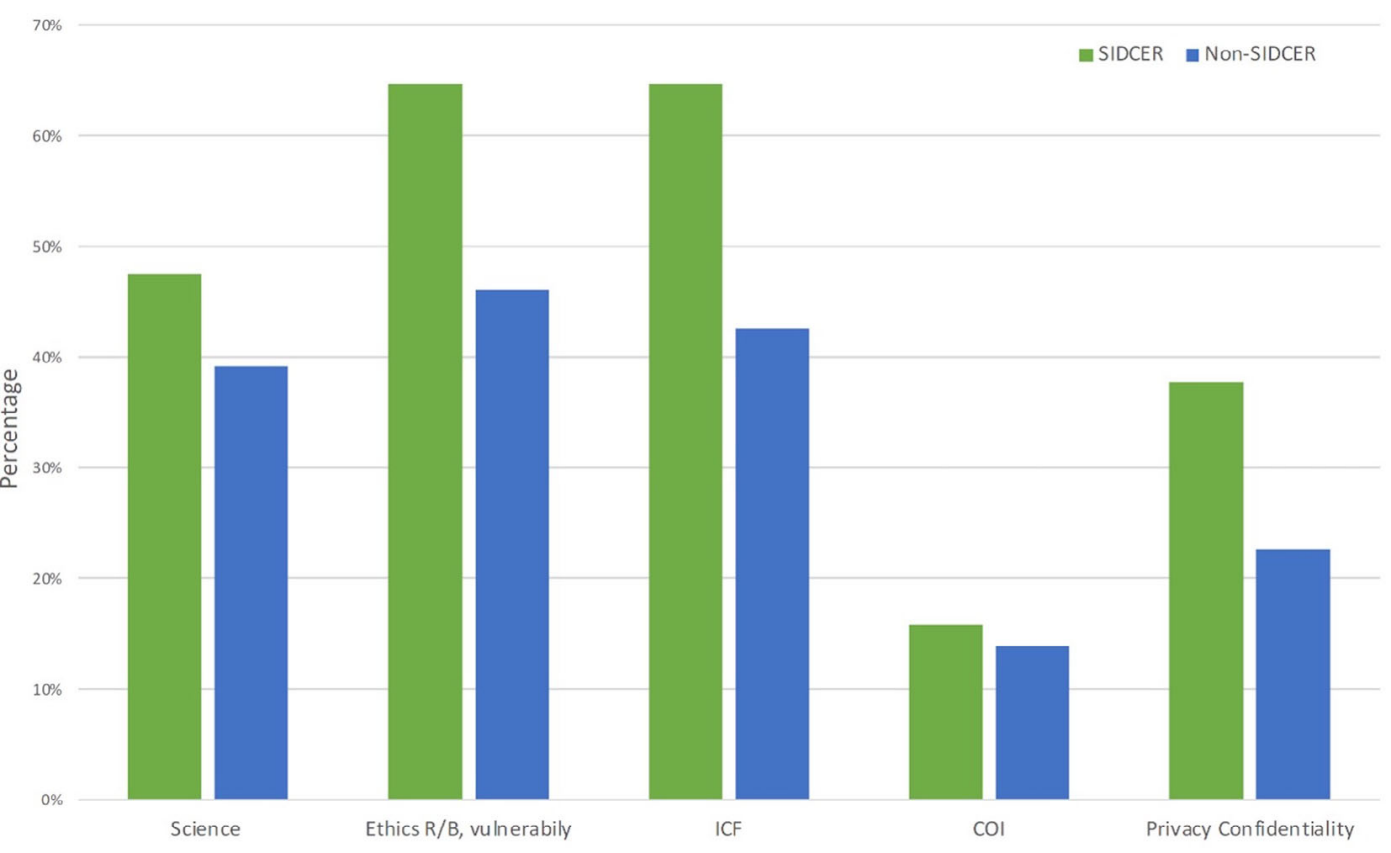
Challenges identified by research ethics committee chairs/secretaries in reviewing COVID-19 protocols.

Although both SR-RECs and NSR-RECs identified challenges in the review of the scientific methodology, ethical issue, ICF, CoI, and privacy and confidential issues ( Figure 5), more SR-RECs identified challenges in the review of risk/benefit and vulnerability (0.010), ICF (0.002), and privacy and confidentiality issues (P = 0.020).

### Training needs for reviewing COVID-19 protocols

**Figure 6.**
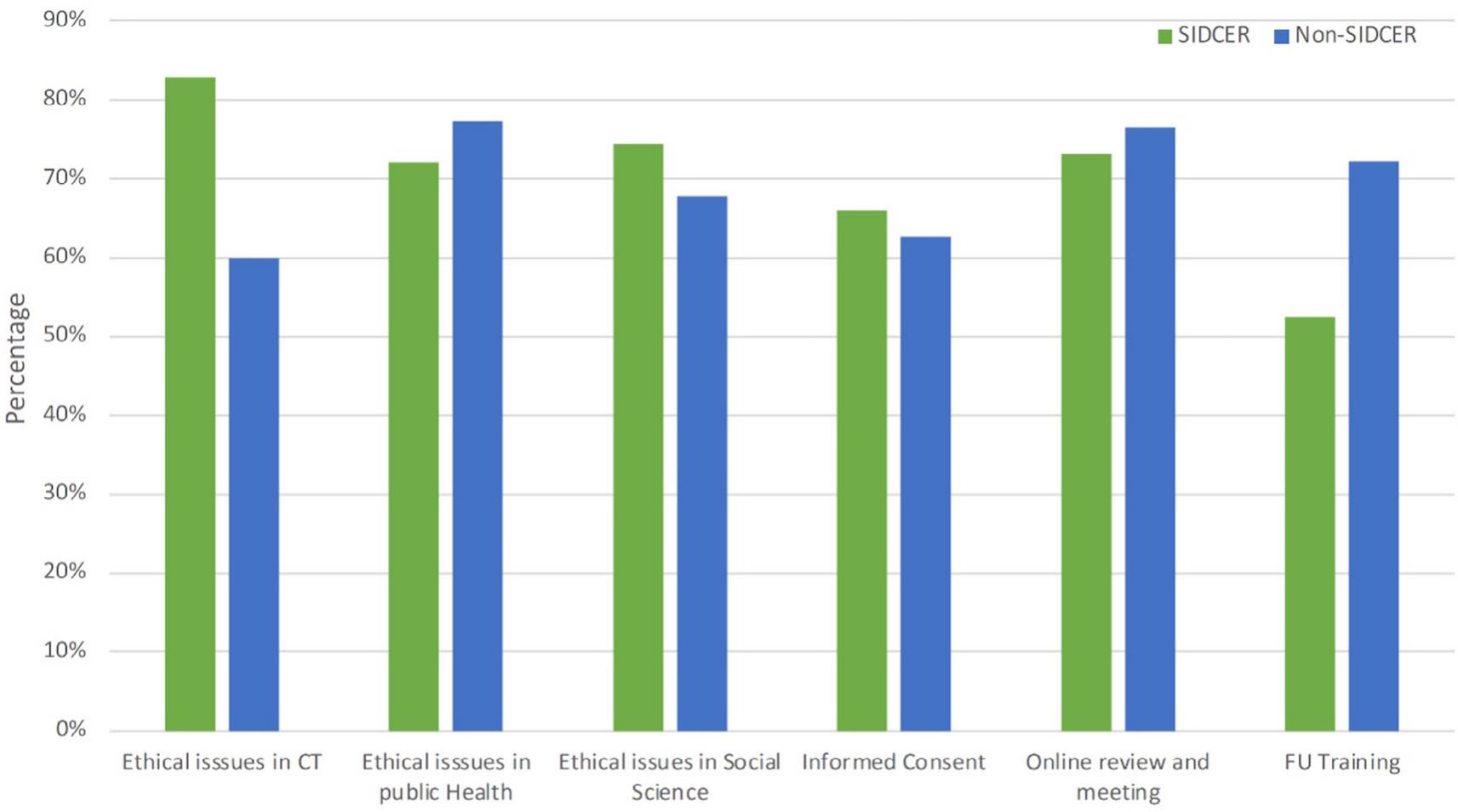
Training recommended by research ethics committee chairs and secretaries.

Similar training needs demanded by the chairs/secretaries of SR-RECs and NSR-RECs are shown in Figure 6. However, the need for training on specific ethical issues related to clinical trials was found to be significantly higher in SR-RECs when compared with NSR-RECs (P = 0.001).

## Discussion

### The composition of Asian RECs

The results from the survey suggested that the RECs in Asian countries generally had satisfactory membership requirements including a multidisciplinary composition as required by international guidelines such as those provided by WHO, ^14^ the Council for International Organizations of Medical Sciences (CIOMS), ^15^ and The International Council for Harmonisation of Technical Requirements for Pharmaceuticals for Human Use (ICH) Good Clinical Practice (ICH GCP). ^16^ The members of these RECs are typically composed of medical doctors/dentists, nurses, pharmacists, biomedical researchers, lawyers, bio-statisticians, social scientists, non-affiliated and laypersons. However, it was noted that some RECs lack laypersons and/or non-affiliated members.

The WHO guidelines emphasize the importance of including laypersons in RECs, as they play a vital role in representing the insights and perspectives of research participants. Additionally, the guidelines highlight the inclusion of non-affiliated members to enhance the independence of the committee. The absence of laypersons and/or non-affiliated members in some RECs may compromise their functionality and not align with the WHO guidelines and the European Medicines Agency (EMA) requirements, which mandate at least one lay and one non-affiliated member. ^14^^,^^17^

The lack of explicit requirements for a layperson in some guidelines such as ICH GCP, may contribute to the absence of laypersons in certain committees. While the ICH GCP only calls for a “non-scientific member”, it is important to note that not all non-scientific members necessarily fulfil the role of a layperson who can effectively represent the perspectives of patients or research participants. ^14^

When comparing the composition of SR-RECs and NSR-RECs, it was found that SR-RECs comply better with international membership requirements by having laypersons and non-affiliated members. Including laypersons and non-affiliated members is crucial to ensure that the voices and perspectives of research participants are considered during the review process and that the decision-making process maintains independence. ^14^

### The RECs’ response to the COVID-19 pandemic

The Asian RECs adjusted well to the restrictions imposed by the government during the COVID-19 pandemic by utilizing online meeting platforms that also enabled them to prioritize the review of COVID-19 protocols by holding more frequent meetings as recommended by WHO as part of the pandemic response. ^18^

To streamline the review process for multi-center COVID-19 protocols and other types of research, most of the REC members in this survey agreed to the use of a joint or single review system. Implementing a joint/single/central REC model was facilitated in Asia due to the harmonization of SOPs among 275 RECs certified under the SIDCER Recognition Program. ^19^ This program fostered trust among SR RECs, enabling them to rely on using joint or single or central REC arrangements during the COVID-19 outbreak. This helped ensure the quality and timely review of multi-center clinical trials. ^18^ It is worth noting that other countries faced challenges in the rapid review of multi-site COVID-19 protocols. South Africa, for example, encountered difficulties related to the operational readiness of their RECs. ^20^ In the United States, using a single IRB has become mandatory for multi-center studies as part of the US Common Rule, aiming to ensure timely review of such studies. ^21^

Overall, the adoption of online platforms, more frequent meetings, and the practice of joint or single review systems have been effective strategies employed by Asian RECs to address the challenges posed by the COVID-19 pandemic and timely review of multi-center research protocols.

### The ability of RECs to identify general ethical issues involving COVID-19 protocols

In general, the RECs in Asia can identify ethical issues in COVID-19 protocols. Most of the REC members classified the COVID-19 protocols as a high-risk type of research due to many uncertainties about the disease, and the vulnerability of the population in low- and middle-income countries. Despite recognizing the vulnerability of COVID-19 patients, REC members agreed that these patients should be included in clinical trials, demonstrating their familiarity with ethical principles outlined in guidelines such as the CIOMS ethical guidelines, which recommend the inclusion of vulnerable participants in health research unless there is a scientific justification for their exclusion. ^22^^–^^25^

More than half of REC members disagreed that drug company employees should be principal investigator in Phase III clinical trials ( Figure 2) indicating their recognition of CoI and the need to manage such conflict. ^26^

Furthermore, research on off-label use of herbal medicine such as andrographolide or repurposed drugs like hydroxychloroquine or ivermectin was common during the pandemic. ^27^^,^^28^ In this survey, many REC Members agreed that the use of herbal medicine and off-label drugs should undergo clinical trials subject to REC review to gather evidence on their safety and efficacy for COVID-19. It was acknowledged that the dose and usage of these substances may differ from their established indications, which could lead to adverse side effects. ^29^^–^^31^

The regular RECs training provided by the SIDCER network, which was established by the WHO special program in Tropical Disease Research (TDR) in 2001, has significantly contributed to the capacity-building of these RECs in Asia regardless of whether they were NSR-RECs or SR-RECs. The SIDCER network conducts regular research ethics training in FERCAP member countries in collaboration with local country fora in the Philippines, Indonesia, India, Sri Lanka, Malaysia, Nepal, Taiwan, and Thailand. ^19^ This demonstrates the importance of active networks of RECs across regions for sharing information, resources, and expertise, and it serves as a strategy to enhance readiness for future public health emergencies.

Overall, the efforts to strengthen REC capacity through training and networking have been instrumental in ensuring that ethical considerations are addressed effectively in COVID-19 research in Asia.

### Requirement of post-approval review

The important responsibilities of RECs are to scrutinize scientific soundness in research protocols that seek to generate new information and novel interventions to control the pandemic and ensure that the researchers uphold ethical principles to protect human participants. ^26^

In addition to the initial review and approval of research protocols, RECs are responsible for monitoring researcher compliance with the approved protocols. This includes reviewing remote patient monitoring reports submitted by research teams and ensuring that researchers are updated about changes in prevention and treatment guidelines as new COVID-19 variants emerge. Post-approval review is essential to ensure the ongoing benefit and safety of research participants.

While both SR-RECs and NSR-RECs reported conducting post-approval reviews, it was found that SR-RECs had more experience in reviewing protocol violations. This may be attributed to the fact that SR-RECs have undergone assessment regularly through the SIDCER Recognition Program, which includes evaluation of the REC’s post-approval monitoring process. This recognition process provides assurance that SR RECs have robust post-monitoring activities in place.

Additionally, SR-RECs often review more product development protocols, especially those supported by pharmaceutical companies. In these cases, there is typically better monitoring of GCP compliance as part of the sponsor’s responsibility; as a rule, the sponsor ensures that the investigators report all deviations/violations to the RECs for review. ^16^

### The RECs call for training on specific COVID-19 issues

Although both SR-RECs and NSR-RECs identified issues encountered with COVID-19, they still expressed the need for further training to effectively analyze and address the unique ethical challenges presented by COVID-19 protocols. The pandemic introduced specific ethical issues that were not encountered before ^32^^–^^35^ and therefore additional training is crucial to ensure the scientific and ethical validity of research.

COVID-19 was a novel disease that spread rapidly and had severe health consequences, including death and serious health conditions. The uncertainty surrounding its origin, transmission, and evolution, combined with the limited availability of treatment and prevention options, posed significant challenges for the scientific community. Researchers and scientists were under pressure to conduct research and find rapid solutions to contain the spread of the virus and mitigate its impact. ^36^^,^^37^ While the urgency of the situation called for expedited research, it was crucial that COVID-19 research adhered to scientific standards and ethical compliance.

New ethical challenges arose due to the public health emergency environment, where authorities imposed the movement restrictions. COVID-19 patients were quarantined at home or in hospitals, and some were in intensive care, making it more difficult to obtain informed consent using traditional practices. ^33^^,^^34^^,^^38^^,^^39^ To adapt to these challenges, RECs faced the task of approving various forms of informed consent, including electronic consent, telephone consent, consent obtained through legally acceptable representatives, oral consent, waivers of signature, and other mechanisms that ensured consent was truly obtained, as required by the RECs. ^34^^,^^39^

To navigate these challenges and ensure that research is conducted with scientific and ethical integrity, REC members require specific training. This training can cover various aspects, such as updates on evolving guidelines and regulations, and enhancing skills in analyzing complex study designs and methodologies.

By providing REC members with targeted training, they can gain the necessary knowledge and tools to effectively assess and address the ethical challenges specific to COVID-19 research. This training helps to promote consistency, rigor, and ethical compliance during the review process, ultimately safeguarding the rights, welfare, and well-being of research participants.

### Challenges in reviewing COVID-19 protocols

The REC members indicated that study design was one of the top challenges in reviewing COVID-19 protocols. In other studies, study design has also been raised as one of the challenges in COVID-19 reviews. ^33^^,^^34^^,^^40^ Adaptive design has been introduced in many COVID-19 clinical trials to enhance the speed of product development. Although the advantages of the adaptive design have been accepted in many scientific communities, it was difficult for RECs to review them as risk/benefit ratio changed when methods, data analysis and products were modified during the course of a study. ^41^^,^^42^ To address these challenges, REC members must familiarize themselves with different study designs, particularly adaptive design. By gaining knowledge and understanding of various study designs, RECs can be better prepared for future epidemics or public health emergencies where quick identification of new treatments is crucial to combat the disease outbreak.

Although both SR-RECs and NSR-RECs identified several challenges in the review of protocols during the pandemic, SR-RECs are better equipped than NSR-RECs in reviewing COVID-19 and investigational medicinal product development protocols. One contributing factor is that SR-RECs have undergone assessment regularly through the SIDCER recognition program. ^19^ The program evaluates various aspects of the REC’s review practice. The assessment includes the appropriateness of the composition of the REC, its operational procedures, and the thoroughness and completeness of the scientific and ethical review protocols. The program ensures that SR-RECs adhere to international ethical guidelines and the ICH GCP in their composition, operation and review processes. By undergoing this assessment, SR-RECs have proven their commitment to maintaining high scientific and ethical review standards. They have demonstrated their capability to effectively manage the review of COVID-19 and investigational product development studies comprehensively and rigorously. This recognition program helps enhance the capacity of SR-RECs and instills confidence in their ability to conduct ethical reviews of research protocols related to public health emergencies like COVID-19.

### Limitation of the study

Most of the participants who responded to the questionnaires were in the medical field and thus may not adequately represent the perspectives of the non-medical REC members. The number of participants was not equal for each country, as well as the SR-RECs and NSR-RECs. The questionnaires were in English thus some participants may not exactly understand the questions and skipped some questions.

## Conclusions

The COVID-19 pandemic created an opportunity to highlight the importance of competent RECs that developed with the assistance of existing local research ethics networks under SIDCER-FERCAP in Asia. The Asian RECs responded to the challenges of the COVID-19 outbreak by adopting online platforms, more frequent meetings, and the use of joint or single review systems to ensure timely review.

During the COVID-19 outbreak, RECs in countries with SIDCER networks generally had a basic understanding of REC operations and ethical principles when reviewing protocols related to the pandemic. However, specific training may have been necessary to enhance the quality of their reviews.

The SR-RECs played a significant role in reviewing COVID-19 protocols, including those related to product development. These RECs effectively managed the unique challenges posed by the pandemic while conducting ethics reviews.

To further strengthen the capacity for future public health emergencies, one strategy is to establish and maintain active networks of ethics committees across regions. These networks facilitate the exchange of new information, resources, and best practices among RECs. By sharing knowledge and collaborating, these RECs can enhance their preparedness and readiness to respond effectively to similar public health emergencies in the future. This collaborative approach promotes harmonized procedure, consistency and high-quality ethics review, ultimately protecting the rights and welfare of research participants.

## Consent

Implied informed consent for publication of the participants’ details was obtained from the participants.

## Data Availability

Figshare: Underlying data for ‘The readiness of the Asian research ethics committees in responding to the COVID-19 pandemic: A multi-country survey’, https://doi.org/10.6084/m9.figshare.24261226.v5. ^13^ This project contains the following underlying data:
• Data file: TDR EC survey 2020 AUG 3 2022.xlsx - Sheet 1 “EC Member”: Survey responses of participants who are REC members. - Sheet2 “COVID Review”: Isolation and processing of the REC members’ survey responses regarding the operation of the members’ respective RECs with regards to COVID-19 protocols and the perception of EC members on ethical issues of COVID-19 research. - Sheet 3 “COVID Review Chart”: Reformatted data from the “COVID Review” sheet to display the processed data as stack bar charts. - Sheet 4 “Chair”: Survey responses of participants who are REC chairs/secretaries. - Sheet 5 “Affiliation”: Isolation and processing of the REC chairs/secretaries’ survey responses regarding the institution type/affiliation of their respective RECs in order to compare the affiliations of SR-RECs versus NSR-RECs. - Sheet 6 “Chair Composition”: Isolation and processing of the REC chairs/secretaries’ survey responses regarding the respondents’ role in their respective RECs in order to compare the compositions of SR-RECs versus NSR-RECs. - Sheet 7 “# Prot Rev”: Isolation and processing of the REC chairs/secretaries’ survey responses regarding the number of protocols reviewed by the respondents’ respective RECs in order to compare the number of protocols reviewed by SR-RECs versus NSR-RECs. - Sheet 8 “Issues”: Isolation and processing of the REC chairs/secretaries’ survey responses regarding the issues reviewed by the respondents’ respective RECs in order to compare the issues reviewed by SR-RECs versus NSR-RECs. - Sheet 9 “Training”: Isolation and processing of the REC chairs/secretaries’ survey responses regarding the training needs of the respondents’ respective RECs in order to compare the training needs of SR-RECs versus NSR-RECs. • Supplementary Figure 1: Participant Distribution.jpg • Supplementary Figure 2: The Roles-Expertise of Participants in REC.jpg • Supplementary Figure 3: Review Function of Participants in REC.jpg • Supplementary Figure 4: SOPs.jpg • Supplementary Figure 5: Frequency of REC Meeting.jpg • Supplementary Figure 6: Experience of Members in Reviewing Types of Protocols.jpg • Supplementary Figure 7: Elements Reviewed by REC when Reviewing COVID Protocols.jpg • Supplementary Figure 8: Challenges Identified by REC Members in Reviewing COVID-19 Protocols.jpg • Supplementary Figure 9: Training Needs when Reviewing COVID-19 Protocols.jpg Figshare: Extended data for ‘The readiness of the Asian research ethics committees in responding to the COVID-19 pandemic: A multi-country survey’, https://doi.org/10.6084/m9.figshare.24261226.v5. ^13^ This project contains the following extended data:
• IRB Covid Survey Questionnaire.pdf • EC-IRB information version 2.docx Data are available under the terms of the Creative Commons Attribution 4.0 International license (CC-BY 4.0)
