# Supplementary material for: The readiness of the Asian research ethics committees in responding to the COVID-19 pandemic: A multi-country survey
Source: F1000Res. 2024 Jan 8;13:19. [Version 1] doi: 10.12688/f1000research.143138.1 (PMC11333877; doi:10.12688/f1000research.143138.1)
Supplement: Peer review history

# Reviewer report: Reviewer response for version 1

**DOI:** 10.5256/f1000research.156768.r306967

**Published:** 2024-08-19

**Authors:** Paul Ndebele

**Affiliations:** 1 The George Washington University, Washington, USA

The study assessed the readiness of RECs in selected Asian countries in responding to the covid-19 pandemic. 

 1. The introduction section is outdated.  Covid-19 is no longer new and the RECs faced most of these challenges during the early days of the pandemic.  The introduction should be recast to show that the authors were reflecting on the situation in 2020. 

 2. It is not clear how the respondents were invited.

 3.  It is not clear how informed consent was obtained.

Is the work clearly and accurately presented and does it cite the current literature?

Yes

If applicable, is the statistical analysis and its interpretation appropriate?

Yes

Are all the source data underlying the results available to ensure full reproducibility?

Yes

Is the study design appropriate and is the work technically sound?

Yes

Are the conclusions drawn adequately supported by the results?

Yes

Are sufficient details of methods and analysis provided to allow replication by others?

Partly

Reviewer Expertise:

Research ethics

I confirm that I have read this submission and believe that I have an appropriate level of expertise to confirm that it is of an acceptable scientific standard.

# Reviewer report: Reviewer response for version 1

**DOI:** 10.5256/f1000research.156768.r298798

**Published:** 2024-08-19

**Authors:** Emma Cave

**Affiliations:** 1 Durham Law School, Durham, UK

This interesting and important paper reports on two connected online surveys across 7 Asian countries in 2021. The research, which was itself subject to research ethics committee (REC) approval in each country, sought to capture the experiences, responses and challenges of RECs to COVID-related research protocols across 332 RECs. It offers a range of original insights and is significant in aiding understanding of the state of the REC approvals systems in Asia and of the research challenges that a global public health emergency can bring. The paper demonstrates the importance of RECs being operationally ready to play their important role in upholding ethical standards whilst facilitating scientifically robust and ethical research, and sets out some of the ways this goal can be achieved. The research is useful in planning for future pandemics and also in considering variation and similarities in composition, role and function of RECs across the different countries.

 Points to consider

1. The survey was carried out in 2021, but in my opinion the article should be updated so that statements made about the current status of the disease are accurate to a 2024 audience. For example, the first two lines stating that the disease is ‘new’ and that there are limited vaccines available is of questionable accuracy in July 2024. The introduction should situate the research in relation to  where we are now as well as where we were in 2021 when the survey was conducted.
2. The introduction could usefully say what is novel about COVID from the perspective of ethics committees. This is mentioned on p 11, but the introduction could set out why the authors considered it to be different and therefore worthy of the survey. Was it the nature of the research (eg clinical, public health), the ethical issues it raises of conducting it (eg human challenge trials etc), the proliferation of new guidelines, the importance of timeliness (new expedited review processes), ways of conducting meetings (eg online) etc, or a range of these issues that prompted the research?
3. For a global audience, it would also be useful for the Introduction to give a little more context on the ethics networks (SIDCER) and regulatory system/s across the 7 countries chosen and how consistent they are in approach. What are the key differences between an SR-REC and a NSR-REC? A conclusion is that the networks need to be strengthened so it would be useful to set out the baseline – what was the state of the networks when the survey was conducted?
4. It would be helpful to say something about whether there was overlap in the data captured by the two surveys and how this was taken into account (eg to avoid double counting).
5. Page 5 ‘most members reviewed on scientific/technical issues …and [some] members reviewed only Informed Consent forms’. Could the authors make clearer whether this is about apportionment of work within the ethics committees (eg some members focus on IC and some on technical issues), or a claim that the ethics committees only review technical issues and some only review IC.
6. I thought the point about variation on SOPs was very interesting. Do the authors have a view as to whether standardisation would be beneficial and if so might it be worth commenting in the conclusions?

Is the work clearly and accurately presented and does it cite the current literature?

Partly

If applicable, is the statistical analysis and its interpretation appropriate?

I cannot comment. A qualified statistician is required.

Are all the source data underlying the results available to ensure full reproducibility?

Yes

Is the study design appropriate and is the work technically sound?

Yes

Are the conclusions drawn adequately supported by the results?

Yes

Are sufficient details of methods and analysis provided to allow replication by others?

Partly

Reviewer Expertise:

I am a law academic who has been involved in related research in the UK. I am not an expert in empirical research methods and so the focus of this review is on the presentation of the results rather than its scientific validity.

I confirm that I have read this submission and believe that I have an appropriate level of expertise to confirm that it is of an acceptable scientific standard.
